# Crosstalk between Acidosis and Iron Metabolism: Data from In Vivo Studies

**DOI:** 10.3390/metabo12020089

**Published:** 2022-01-18

**Authors:** Raêd Daher, Nicolas Ducrot, Thibaud Lefebvre, Sofia Zineeddine, Jérome Ausseil, Hervé Puy, Zoubida Karim

**Affiliations:** 1 Centre de Recherche sur l’Inflammation (CRI), Université de Paris, INSERM, CNRS, F-75018 Paris, France; raed.daher@hotmail.fr (R.D.); nicolas.ducrot@inserm.fr (N.D.); thibaud.lefebvre@inserm.fr (T.L.); sofia.zineeddine@inserm.fr (S.Z.); herve.puy@inserm.fr (H.P.); 2 Centre Français des Porphyries, Hôpital Louis Mourier, APHP, Nord-Université de Paris, F-75014 Colombes, France; 3 Institut Toulousain des Maladies Infectieuses et Inflammatoires (Infinity), Université de Toulouse, INSERM, CNRS, F-31024 Toulouse, France; jerome.ausseil@inserm.fr

**Keywords:** acidosis, acid secretion, ATP4, hepcidin, iron metabolism

## Abstract

Iron absorption requires an acidic environment that is generated by the activity of the proton pump gastric H(+)/K(+)ATPase (ATP4), expressed in gastric parietal cells. However, hepcidin, the iron regulatory peptide that inhibits iron absorption, unexpectedly upregulates ATP4 and increases gastric acidity. Thus, a concept of link between acidosis and alterations in iron metabolism, needs to be explored. We investigated this aspect in-vivo using experimental models of NH4Cl-induced acidosis and of an iron-rich diet. Under acidosis, gastric ATP4 was augmented. Serum hepcidin was induced and its mRNA level was increased in the liver but not in the stomach, a tissue where hepcidin is also expressed. mRNA and protein levels of intestinal DMT1(Divalent Metal Transporter 1) and ferroportin were downregulated. Serum iron level and transferrin saturation remained unchanged, but serum ferritin was significantly increased. Under iron-rich diet, the protein expression of ATP4A was increased and serum, hepatic and gastric hepcidin were all induced. Taken together, these results provide evidence of in-vivo relationship between iron metabolism and acidosis. For clinical importance, we speculate that metabolic acidosis may contribute in part to the pathologic elevation of serum hepcidin levels seen in patients with chronic kidney disease. The regulation of ATP4 by iron metabolism may also be of interest for patients with hemochromatosis.

## 1. Introduction

Iron from the diet goes through several processes before being absorbed across the epithelium of the proximal small intestine, and the regulation of iron absorption is essential for maintaining iron levels in the body within a physiologically defined range.

To be absorbed, diet ferric iron (Fe(III)) is rapidly reduced to the ferrous form (Fe(II)) by the apical reductase Duodenal cytochrome B (Dcyt B) [1] and transported by the duodenum through the Divalent Metal Transporter 1 (DMT1) located at the apical membrane. Fe(II) is then exported into the blood through the iron exporter Ferroportin (FPN) located at the basolateral membrane of the enterocyte, where it is oxidized again into Fe(III) and bound to transferrin (Tf) before distribution to other tissues [2,3,4,5]. However, this availability of iron is largely dependent on the intraluminal gastric acid secretion. Low pH prevents the polymerization and the precipitation of Fe(III) [6] and promotes its reduction to Fe(II) to be taken up by DMT1. In addition, DMT1 operates as a symporter of divalent metal ions and H^+^, while the proton electrochemical potential gradient is the driving force for the metal transport [4].

Gastric acid is secreted by the parietal cells [7,8], which secrete HCl by the electroneutral ATP-dependent pump H+/K+ ATPase (also named ATP4). ATP4 consists of 2 subunits, ATP4A that contains the catalytic site of the pump [9], and ATP4B, which protects the pump from degradation and is necessary for targeting to the plasma membrane [10]. Patients with hypochloridria, suffer from an iron-deficiency anemia caused by decreased iron absorption [11] and treatment with proton pump inhibitors (PPIs), often used against gastric acidity associated disorders, frequently causes anemia associated with iron absorption defect [12]. Treatment of patients with hereditary hemochromatosis with PPIs significantly reduced the number of phlebotomies required to prevent excess iron overload [13,14]. Finally, a missense mutation in ATP4A was described to be responsible for a microcytic hypochromic anemia in a sublityc mouse model [15].

The liver-derived peptide hepcidin acts as a central regulator of body iron homeostasis, including iron absorption and iron efflux from the sequestering/recycling reticuloendothelial system [16,17]. In macrophages, hepcidin limits iron export by binding to FPN leading to its internalization and degradation [18,19]. However, in the absorptive cells, we and others have found that hepcidin reduces iron absorption by acting primary on apical iron uptake and DMT1 importer [20,21,22,23]. Besides the liver that is the main site of hepcidin synthesis, several other organs were shown to produce this peptide although at lesser extent [24,25,26,27,28]. Hepcidin was shown to localize in gastric parietal cells and to regulate acid secretion [24]. Hepcidin was also shown to be produced in epithelial cells of renal distal nephron and hepcidin knockout (KO) mice was found to exhibit significant reduction in ATP4A expression, leading to the significant alkalization of urine that was restored after exogenous hepcidin treatment [29]. However, the relevance of this regulation of ATP4 by hepcidin and, conversely, a possible regulation of hepcidin by acidosis has not yet been fully determined.

In the present study, we investigated the reciprocal link between acidic pH and hepcidin-regulated iron balance in a mouse model of acidosis, as well as in an iron-rich mouse model overexpressing hepcidin.

## 2. Results

### 2.1. Both ATP4A and ATP4B Are Stimulated by Acidosis

**Figure 1:**
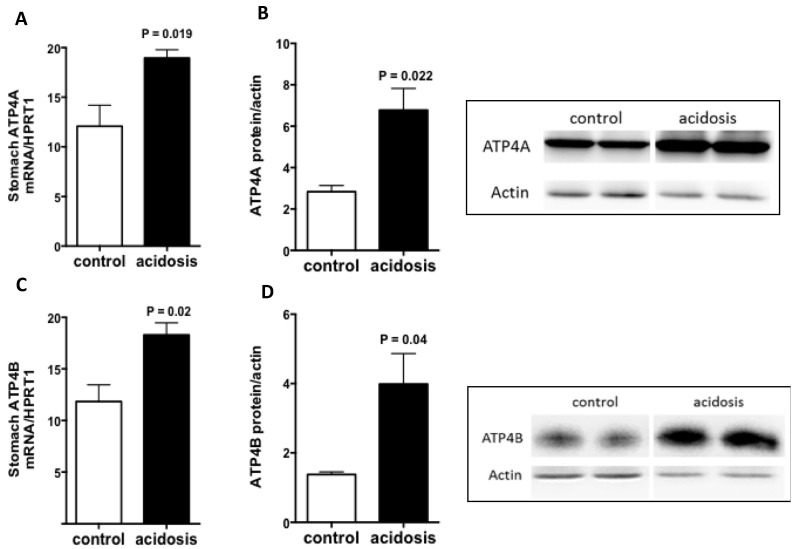
Regulation of ATP4A and ATP4B expression by acidosis in the stomach. (**A**,**C**): quantification of the mRNA levels of ATP4A and ATP4B in the stomachs of control and acidosis mice, respectively. (**B**,**D**): left panel: Representative western blot images of ATP4A and ATP4B proteins, and right panel is the quantification of the ratio of ATP4A on actin signals, respectively. Data are mean ± sem, *n* varies between 3 and 4 mice for each group.

Stomach samples obtained from control and acidosis mice were explored for an expected increase in ATP4. Quantitative Reverse Transcriptase PCR (RT-PCR) and Western Blot experiments were performed, and the analyses revealed a significant increase in the transcript level of Atp4A (*p* = 0.019) (Figure 1A) and its protein expression (Figure 1B) in the stomachs of acidosis mice compared with control mice. The ATP4B, which is responsible for targeting and stabilizing ATP4A at the plasma membrane, was also regulated by acidosis in the same manner. The results show a significant increase in Atp4B transcript level (*p* = 0.02) (Figure 1C) as well as an augmentation in its protein expression (Figure 1D) in the stomachs of acidosis mice. Using a mouse experimental model of acidosis, the results demonstrate that gastric ATP4 expression was increased as described in previous studies [30,31], confirming that the current model mimic metabolic acidosis, as we have already shown in a rat [32].

### 2.2. Hepcidin Expression Is Increased in Acidosis Condition

**Figure 2:**
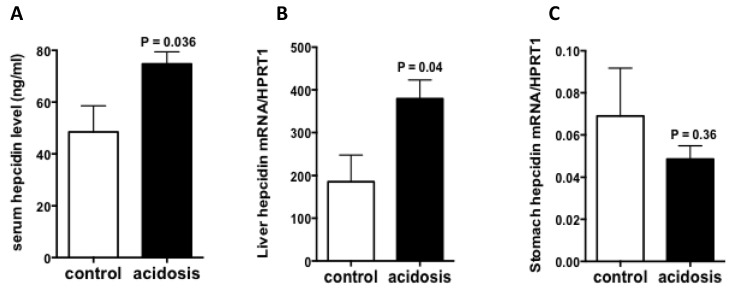
Regulation of hepcidin expression by acidosis. (**A**): Serum hepcidin measurement by LC-MS/MS. (**B**,**C**): Quantification of hepcidin mRNA levels in the liver and stomach of control and acidosis mice. Data are mean ± sem, *n* = 8 mice of each group in (**A**) and *n* = 4 mice of each group in (**B**,**C**).

To see if acidosis also regulates the expression of hepcidin, serum hepcidin was first measured by LC-MS/MS(Liquid chromatography–mass spectrometry/mass spectrometry), and the results showed increased levels in acidosis mice comparing to control mice (Figure 2A). Then, the mRNA expression of hepcidin was quantified RT-qPCR experiments in stomach and liver samples of acidosis and control mice. The results showed that the mRNA levels of hepatic hepcidin was significantly increased (Figure 2B), however, gastric hepcidin mRNA levels remained unchanged in acidosis condition relative to control (Figure 2C).

### 2.3. Both Duodenal Iron Transporters DMT1 and FPN Are Downregulated under Acidosis Condition

**Figure 3:**
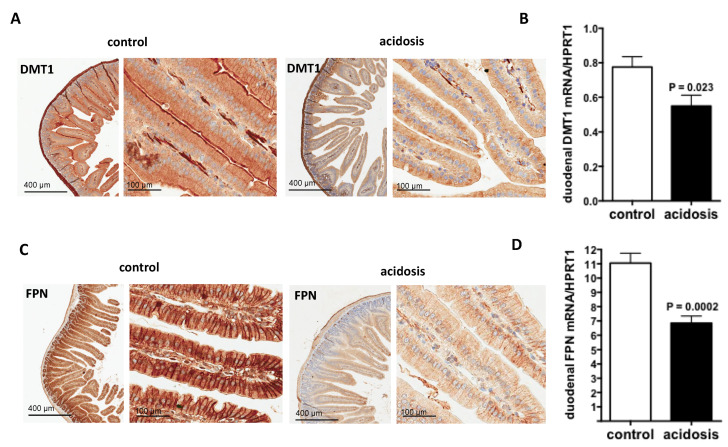
Regulation of duodenal iron transporters DMT1 and FPN by acidosis. (**A**,**C**): Representative images of immunohistochemistry staining of DMT1 and FPN in duodenal sections of control and acidosis mice, respectively. Data are explored in *n* = 8 mice for each group. (**B**,**D**): quantification of the mRNA levels of DMT1 and FPN in duodenums of control and acidosis mice, respectively. Data are mean ± sem, *n* = 8 mice for each group.

DMT1 transporter is responsible for inorganic iron uptake by the absorptive enterocytes and ferroprtin (FPN), the solely iron exporter for enterocytes and for all living cells. Therefore, their expression patterns were investigated during acidosis by immunohistochemistry staining on duodenal sections of the two groups of mice. For DMT1, the specific apical staining in microvilli was considerably reduced in acidosis mice compared with control mice (Figure 3A). For FPN, the staining was basolateral and was also reduced in acidosis mice compared with control mice (Figure 3C).

DMT1 and FPN transcript levels were studied by quantitative RT-PCR performed on duodenum samples of both acidosis and control mice. The data obtained indicate that the mRNA-expression levels of both Dmt1 and Fpn were decreased under acidosis conditions (*p* = 0.023 and *p* = 0.0002 versus controls, respectively) (Figure 3B,D, respectively).

### 2.4. Regulation of Iron Status by Acidosis

**Figure 4:**
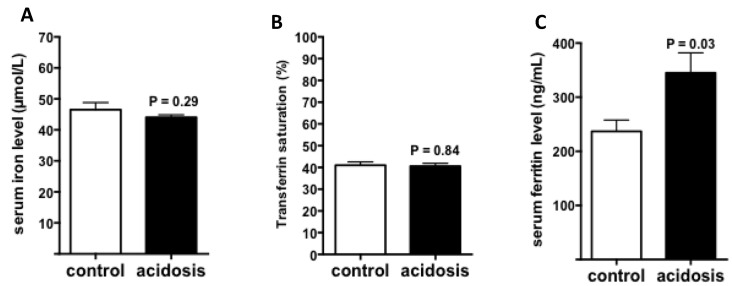
Regulation of iron status by acidosis. Serum iron parameters of control and acidosis mice were measured using an AU400 automate. (**A**): Serum iron level. (**B**): Transferrin saturation. (**C**): Serum ferritin level. (*n* varies between 6 and 8 mice).

To further study the impact of acidosis on systemic iron status, iron, and ferritin levels, as well as transferrin saturation, were measured in serum of control and acidosis mice. The results showed that both serum iron level and transferrin saturation remained unchanged in acidosis mice compared with the control group (Figure 4A,B). However, serum ferritin levels were significantly increased under acidosis conditions (*p* = 0.03 versus controls) (Figure 4C).

### 2.5. ATP4A Expression Is Stimulated in Iron-Rich Diet

**Figure 5:**
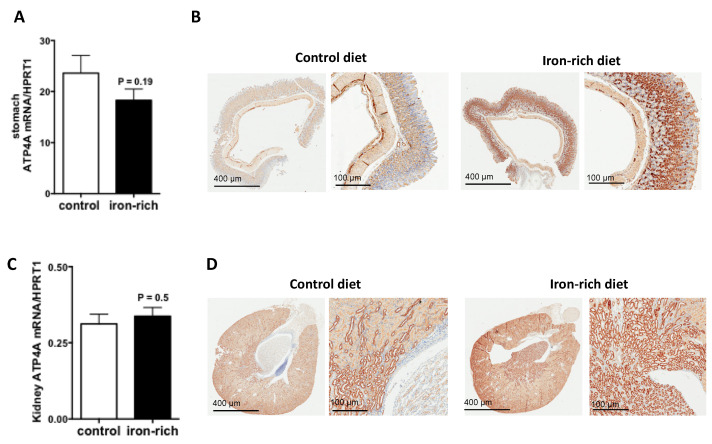
Regulation of ATP4A by iron in the stomach and kidney. (**A**,**C**): quantification of the mRNA levels of Atp4 in stomach and kidney sections of control and acidosis mice, respectively, (**B**,**D**): Representative images of immunohistochemistry staining of ATP4A in stomach and kidney sections of control and acidosis mice, respectively. Data are explored in *n* = 8 mice for each group.

**Figure 6:**
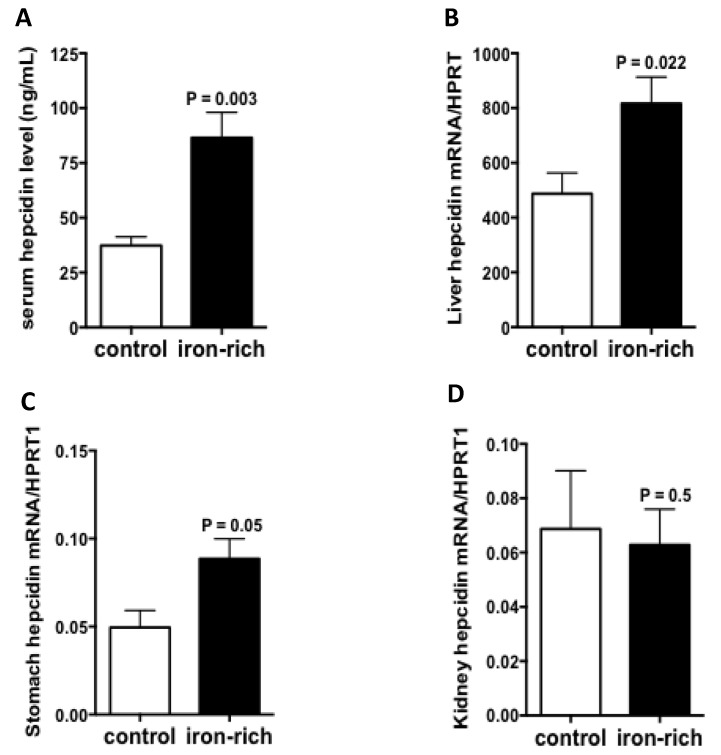
Regulation of gastric and renal hepcidin by iron. (**A**): Serum hepcidin levels measured by LC-MS/MS in control and iron-rich feeding mice. (**B**–**D**): Hepcidin mRNA quantification in the liver, stomach and kidneys of control and iron-rich feeding mice, respectively. Data are mean ± sem, normalized by the housekeeping gene Hprt1. Data are explored in *n* = 7 mice for each group.

Since ATP4A was reduced in hepcidin knockout mice both in stomach and kidney [24,29], we investigate its regulation by iron-rich diet, a condition where hepatic hepcidin is induced. The results of quantitative RT-PCR experiments showed that the transcript levels of ATP4A remained unchanged but the immunostaining experiments showed that the expression of ATP4A protein was strongly stimulated under conditions of iron-rich diet relative to control diet (Figure 5A,B). Similar data were also observed in the kidney (Figure 5C,D). Serum hepcidin was as expected, significantly augmented by the iron-rich diet (*p* = 0.003) (Figure 6A). The transcript levels of hepatic and gastric hepcidin transcripts were both increased under iron-rich conditions compared with control conditions (*p* = 0.022 and *p* = 0.05, respectively) (Figure 6B,C). In contrast, the iron-rich diet did not affect the mRNA expression of renal hepcidin compared with control mice (Figure 6D).

## 3. Discussion

Dysregulation of iron homeostasis represents one of the key-player in anemia of chronic diseases including chronic kidney diseases where functional iron deficiency is persistent, leading to reduced iron availability for erythropoiesis and subsequent hyporesponsiveness to iron therapies and resistance to erythropoiesis-stimulating agents (ESAs). This functional iron deficiency is largely attributed to elevated levels of serum hepcidin, an elevation that is multifactorial due at least in part to diminished renal clearance and an inflammatory state [30,31,32,33].

Using a mouse experimental model of acidosis, the results demonstrate that acidosis is also an additional factor to be taken into account for hepcidin elevation in theses clinical contexts. Indeed, hepcidin was increased at mRNA and protein levels with additional increased of serum ferritin levels, suggestive of an iron retention in tissue stores. The mechanism by which metabolic acidosis may promote hepcidin in liver is not yet understood but reports have shown in human hepatoma cell lines that acidic medium was able to augment hepcidin expression via stabilization of its transcript [34]. Interestingly, stabilization of the hepcidin transcripts has also been demonstrated in vivo, in the context of non-alcoholic fatty liver disease (NAFLD), a condition that can develop as a result of diet-induced acid load [35].

In the duodenum, the mRNA and protein expression patterns of both DMT1 and FPN were reduced in acidosis conditions. Elevated hepcidin must contribute in this effect since both DMT1 and FPN protein abundances are negatively regulated by hepcidin [19,20]. However, additional factors may also contribute to the decrease in the duodenal DMT1 and FPN mRNA expressions. Since DMT1 activity is dependent to acidic pH, we assume that an increase in iron intake due to increase DMT1 activity by diet-induced acid load must be retro-controlled by the local IRE/IRP system. Indeed, iron regulatory protein IRP1 appears to be active in the intestinal compartment [36] and the 3′-UTR region of DMT1-mRNA contains at least one IRE element, which was recently shown to be active [37,38]. The IRE/IRP system also acts on the expression of the FPN by blunting its translation after binding to the 5′-UTR-IRE of the mRNA [37]. The data show that FPN mRNA is also reduced by acidosis, suggesting an independent mechanism of IRE/IRP. Inflammation that appears in the intestine during acidosis, may be responsible for FPN transcriptional regulation [39] Indeed, inflammation have been shown to directly decrease FPN mRNA expression in a hepcidin-independent manner [40,41,42,43] Thus, overall data suggested that metabolic acidosis disturbed iron homeostasis as showed by elevated serum hepcidin with normal transferrin saturation and increased hepcidin production, a clinical phenotype observed in several chronic diseases with functional iron deficiency.

Studies on iron-rich diet-fed mice showed that the ATP4A protein level was strongly increased in both the stomach and the kidney. The data showed that the increase in protein level of ATP4A in both organs was independent of transcriptional regulation, suggesting translational upregulation. This later may not be due to the IRE/IRP system since a search for IRE sequences failed to identify any element in Atp4A mRNA and predicted three IRE sequences in mouse Atp4B mRNA that are not conserved in humans (data not shown). In addition, the fact that the protein level of ATP4A increased in both organs while hepcidin increased only in the stomach but not in the kidney, suggests that this upregulation of ATP4A is dependent on systemic hepcidin rather than a local synthesis of this peptide. The upregulation of ATP4A by iron rich diet, which may potentially reinforce iron intake during high iron-rich diet, remained paradoxical with regards of iron overload prevention. Nevertheless, these results could support the studies showing that the treatment of patients with hereditary hemochromatosis by Proton Pump Inhibitors (PPIs) is beneficial for the reduction in iron absorption and stores, which reduces the frequency of phlebotomy therapies [13,14].

In conclusion, the results described in the present study reinforce the hypothesis of the existence of a link between hepcidin and acidosis. Acidosis induces hepatic hepcidin synthesis, and reciprocally, hepcidin overexpression promotes acid secretion in stomach and kidney.

## 4. Materials and Methods

### 4.1. Animals

Five-week-old CBA/J-strain female mice were purchased from the Janvier-Europe laboratory and acclimated in animal facility for a few days. They were housed under specific pathogen-free conditions with strict control of light, temperature (21 °C), and humidity (50–60% relative humidity). Standard food and water were available ad libitum. All of the animal studies were conducted in compliance with EU directives for animal experimentation and were approved by the Ethical Committee of Paris North and The French Minister of Higher Education, Research and Innovation.

### 4.2. Experimental Animal Models

The mouse model of acidosis was used as described by Attmane-Elakeb et al. [44]. Briefly, experimental mice were given 0.28 M NH_4_Cl in distilled drinking water, whereas control mice drank distilled water for 2 weeks. The mouse model of iron rich diet was generated by feeding mice with 2 g iron/kg diet (C1000 control diet supplemented with iron sulfate, GENESTIL (ALTROMIN) F-60420 ROYAUCOURT, France) for 3 weeks. Control mice were feed with standard diet (C1000 control that contains 178 mg iron/kg diet). At the end of the diet, the animals were sacrificed. The liver, kidney, duodenum, and stomach were aseptically removed. Small sections of the duodenum and stomach were prepared for immunohistochemistry, and the epithelial cells were detached in distilled water supplemented with 1× protease inhibitor cocktail (EDTA Complete, Thermo Scientific, Illkirch, France). Samples were then pelleted by centrifugation and stored at −80 °C.

For each experimental model, 2 independent experiments were carried out each using 8 mice (4 controls, 4 treated).

### 4.3. Biochemical Analyses

For serum collection, mice were anesthetized by intraperitoneal injection of Pentobarbital (Ceva Santé Animale, Paris, France), and blood was drawn from the retro-orbital sinus with capillary Pasteur pipettes and derived serum was immediately stored at −20 °C until analysis. Serum non-heme iron, ferritin, and transferrin levels were measured using an AU400 automate (Olympus, Tokyo, Japan). Serum hepcidin was measured using a previously validated LC-MS/MS method [45].

### 4.4. Immunohistochemistry

Immunohistochemistry was carried out using an automated immunohistochemical stainer according to the manufacturer’s guidelines (Bond-Max Autostainer; Leica, Wetzlar, Germany) after dewaxing and rehydrating paraffin sections and antigen retrieval by pretreatment with high temperature at pH 9. After antigen retrieval, tissue sections were immunolabeled with primary antibodies as follows: DMT1 (produced by Biomatik, Wilmington, DE, USA): diluted 1:400, pH 9; FPN (kind gift from Dr. Haile, San Antonio, TX, USA): diluted 1:150, pH 6; ATP4A (Antibodies-online, Aachen, Germany): diluted 1:100, pH 9. Substitution of the primary antibody with phosphate-buffered saline was used as a negative control. Tissue sections were then incubated with the secondary antibody polymer for 10 min (Bond Polymer Refine detection; DS9800; Leica Microsystems, Wetzler, Germany) and developed with DAB-chromogen for 10 min.

### 4.5. Quantitative RT-PCR

Total RNA was isolated from liver samples or cell pellets using RNA-PLUS reagent (MP-Europe) according to the manufacturer’s recommendations. Complementary DNA (cDNA) was synthesized using Maxima First Strand cDNA Synthesis kit (ThermoFisher Scientific, Villebon-sur-Yvette, France) as per the manufacturer’s instructions, using 1 µg total RNA template per sample. Quantitative reverse transcriptase polymerase chain reaction (RT-qPCR) was performed with specific sets of primers and LightCycler 480 SYBR Green I Master (Roche Diagnostics, Mannheim, Germany) and run on a LightCycler 480 Instrument (Roche Diagnostics).

Hamp1, Atp4A, Atp4B, Fpn, Dmt1 and Hprt1 (Hypoxanthine Phosphoribosyltransferase 1) transcripts were amplified with specific primers (Table 1). Hprt1 transcripts were used as an internal control. Standard curves for all of the cited genes were generated from accurately determined dilutions of cDNA. Samples were analyzed in duplicate, and results are reported as the ratio of mean values for the different genes to Hprt1.

### 4.6. Protein Extraction and Immunoblotting (WB)

Stomach and duodenal cells were resuspended in radioimmunoprecipitation assay (RIPA) buffer (150 mM NaCl, 50 mM Tris/HCl pH 7.6, 1% Triton, 0.1% SDS and 26.6 mg/mL AEBSF) supplemented with 1× protease inhibitor cocktail (EDTA Complete, Thermo Scientific, Illkirch, France). The homogenate was incubated in the same buffer for 1 h at 4 °C, and mixed by vortexing every 10–15 min. After centrifugation at 10,000× *g* for 10 min, the supernatant containing total proteins was transferred to a fresh tube and stored at −80 °C until use. Protein concentrations were determined using the Bradford protein assay (BIO-RAD Laboratories, München, Germany). Equal amounts of proteins (30 µg) were separated by electrophoresis on a 10% SDS-polyacrylamide gel. Primary antibodies were used at 1:1000 for anti-DMT1 (produced by Biomatik, Wilmington, DE, USA), anti-ATP4A and anti-ATP4B (Clinisciences, Nanterre, France), and at 1:10,000 for the anti-actin mouse monoclonal antibody (Sigma-Aldrich Fine Chemicals, St. Quentin Fallavier CEDEX, France), which was used as a loading control. Immunoreactive bands were revealed by HRP-conjugated secondary antibodies using Amersham ECL.

### 4.7. Statistical Analysis

Statistical significance was tested with Student’s *t* test. A finding of *p* < 0.05 was considered significant.

## Figures and Tables

**Table 1:** Forward and reverse sequences of genes used for quantitative RT-PCR.

Gene	Forward Sequence	Reverse Sequence
Mouse *Hamp*	CGATACCAATGCAGAAGAGAAGG	TTTGCAACAGATACCACACTGGG
Mouse *Atp4a*	AGCACCAGGCACCATGGGGAAG	CACCAGGGCCAGACCCCAGTT
Mouse *Atp4b*	ACCCCTACACCCCAGACTAC	CCATACACGTCCGGTCTCAA
Mouse *Dmt1*	GGCTTTCTTATGAGCATTGCCTA	GGAGCACCCAGAGCAGCTTA
Mouse *Fpn*	CCCATAGTCTCTGTCAGCCTGC	CCGTCAAATCAAAGGACCAAA
Mouse *Hprt1*	AGCTACTGTAATGATCAGTCAACG	AGAGGTCCTTTTCACCAGCA

## Data Availability

The data is available at INSERM UMR1291.
